# Ring vaccination with rVSV-ZEBOV under expanded access in response to an outbreak of Ebola virus disease in Guinea, 2016: an operational and vaccine safety report

**DOI:** 10.1016/S1473-3099(17)30541-8

**Published:** 2017-12

**Authors:** Pierre-Stéphane Gsell, Anton Camacho, Adam J Kucharski, Conall H Watson, Aminata Bagayoko, Séverine Danmadji Nadlaou, Natalie E Dean, Abdourahamane Diallo, Abdourahmane Diallo, Djidonou A Honora, Moussa Doumbia, Godwin Enwere, Elizabeth S Higgs, Thomas Mauget, Diakite Mory, Ximena Riveros, Fofana Thierno Oumar, Mosoka Fallah, Alhassane Toure, Andrea S Vicari, Ira M Longini, W J Edmunds, Ana Maria Henao-Restrepo, Marie Paule Kieny, Sakoba Kéïta

**Affiliations:** a World Health Organization, Geneva, Switzerland; b Faculty of Epidemiology and Population Health, London School of Hygiene & Tropical Medicine, London, UK; c Centre National d'Appui à la Lutte contre la Maladie, Bamako, Mali; d Department of Biostatistics, University of Florida, Gainesville, FL, USA; e World Health Organization Ebola Vaccine Trial and Compassionate Use Team, Conakry, Guinea; f National Public Health Institute of Health, Monrovia, Liberia; g Division of Clinical Research, National Institute of Allergy and Infectious Disease, Bethesda, MD, USA; h Ebola Response, Ministry of Health, Conakry, Guinea

## Abstract

**Background:**

In March, 2016, a flare-up of Ebola virus disease was reported in Guinea, and in response ring vaccination with the unlicensed rVSV-ZEBOV vaccine was introduced under expanded access, the first time that an Ebola vaccine has been used in an outbreak setting outside a clinical trial. Here we describe the safety of rVSV-ZEBOV candidate vaccine and operational feasibility of ring vaccination as a reactive strategy in a resource-limited rural setting.

**Methods:**

Approval for expanded access and compassionate use was rapidly sought and obtained from relevant authorities. Vaccination teams and frozen vaccine were flown to the outbreak settings. Rings of contacts and contacts of contacts were defined and eligible individuals, who had given informed consent, were vaccinated and followed up for 21 days under good clinical practice conditions.

**Findings:**

Between March 17 and April 21, 2016, 1510 individuals were vaccinated in four rings in Guinea, including 303 individuals aged between 6 years and 17 years and 307 front-line workers. It took 10 days to vaccinate the first participant following the confirmation of the first case of Ebola virus disease. No secondary cases of Ebola virus disease occurred among the vaccinees. Adverse events following vaccination were reported in 47 (17%) 6–17 year olds (all mild) and 412 (36%) adults (individuals older than 18 years; 98% were mild). Children reported fewer arthralgia events than adults (one [<1%] of 303 children *vs* 81 [7%] of 1207 adults). No severe vaccine-related adverse events were reported.

**Interpretation:**

The results show that a ring vaccination strategy can be rapidly and safely implemented at scale in response to Ebola virus disease outbreaks in rural settings.

**Funding:**

WHO, Gavi, and the World Food Programme.

## Introduction

Between 2013 and 2015, more than 28 000 cases of Ebola virus disease were reported across Guinea, Sierra Leone, and Liberia. In response to the epidemic, trials of several candidate vaccines were fast-tracked. In July, 2015, interim results from the Ebola ça Suffit ring vaccination phase 3 cluster-randomised trial^1^ of the rVSV-ZEBOV vaccine in Guinea were published. On the basis of interim analysis, the trial showed 100% (95% CI −74·7 to 100%) vaccine efficacy, with 75·1% (95% CI −7·1 to 94·2%) vaccine effectiveness at the cluster level, including herd immunity of unvaccinated members of clusters.^1^

Guinea was declared Ebola virus disease-free on Dec 29, 2015, and the trial ended on Jan 20, 2016, after the final participants completed the 84-day follow-up. Surveillance for Ebola virus continued, and on March 17, 2016, two new laboratory-confirmed cases of Ebola virus disease were identified in the Nzérékoré prefecture (Guinea) of the inland Guinée Forestière region.^2^

Here, we report on the implementation and impact of the ring vaccination strategy in Guinea during this flare-up. We also evaluate the vaccine safety in different populations and examine the dynamics of transmission at the level of the rings.

## Method

### Study design

Under compassionate use, ring vaccination used a simplified version of the ring Ebola virus vaccination trial protocol^3^: all rings were immediately vaccinated and follow-up of vaccinees was done 30 min after vaccination and on days 3, 14, and 21 (visits on days 42, 63, and 84 were omitted).

We also used a similar operational approach to the one used for the Ebola ring vaccination trial^3^ and the activities were done in accordance with good clinical practice (GCP). Before the confirmation of the first case of Ebola virus disease in this outbreak, a GCP-trained team drawn from members of the Ebola ça Suffit trial was in Conakry (Guinea) preparing for a cohort study on contacts of survivors of Ebola virus disease.

**Research in context**

**Evidence before this study**

No licensed vaccines are available for the prevention of Ebola virus disease or other filovirus infections. The rVSV-ZEBOV candidate vaccine has been reported to be protective and well tolerated in adults in a phase 3 clinical trial. We searched MEDLINE and Embase without language restrictions for articles published from January, 1990, to July 20, 2017, to identify any published report of the use of an Ebola vaccine under expanded access, with the search terms “Ebola virus”, “vaccine”, “children”, “compassionate use”, and “expanded access”. To our knowledge, our manuscript is the first documentation on the use of an Ebola vaccine under expanded access during an outbreak, and is the first to report on the safety profile of vaccinated children at risk of Ebola virus transmission. Therefore, we could not do a detailed systematic review at this point.

**Added value of this study**

Our study illustrates how a ring vaccination strategy can be implemented in a rural setting and in combination with other public health interventions. Furthermore, our study comprises an unprecedented dataset of safety information in children older than 6 years following immunisation. Our results show that a ring vaccination strategy can be rapidly and effectively implemented at scale in response to Ebola virus disease outbreaks in rural settings and across borders.

**Implications of all the available evidence**

Our findings underscore the importance of the ring vaccination strategy to be considered for an Ebola virus disease outbreak response as a public health intervention. Additionally, our results suggest that the ring vaccination approach is compatible with the use of an experimental vaccine under expanded access, and that children older than 6 years should be included in the ring definition. Ring vaccination might have application in the delivery strategy of other vaccine candidates in epidemics of other viral haemorrhagic fevers or other emerging infectious diseases.

Immediately after the confirmation of the first case in March, 2016, and following a request by the national authorities, WHO prepared a protocol for submission to the national regulator (Direction Nationale de la Pharmacie et du Laboratoire) and Guinean Ethics Review Committee (Comité National d'Ethique pour la Recherche en Santé), and the WHO Ethics Review Committee. The request was for authorisation to provide the rVSV-ZEBOV vaccine to contacts of recently confirmed case of Ebola virus disease under expanded access and compassionate use. At the time of this outbreak, rVSV-ZEBOV was still an unlicensed product. Expanded access and compassionate use were unanimously authorised within 48 h.

In parallel, trial personnel were selected on the basis of their skills and role during the trial and flown to Nzérékoré, Guinée Forestière, with support from the World Food Programme. In total, 35 trial staff members were deployed to Guinée Forestière with the aim of being able to implement four rings in parallel.

Once the regulatory and ethical approvals were obtained, a team of social mobilisers engaged with the local authorities and communities to obtain local approval for the team to visit the area and enumerate the ring members. National authorities held meetings with local authorities and leaders to explain the objectives and the known information about the candidate vaccine. Community engagement was an important part of the activity, particularly because the region of Guinée Forestière had been Ebola virus-free for a year before this case and communities were understandably reluctant to accept that Ebola virus disease had returned.

Once community authorisation was obtained, an epidemiological team defined a ring by enumerating the contacts and contacts of contacts of the index case. Contacts were individuals who visited or were visited by the index case after the onset of symptoms; had lived in the same household; or were in close physical contact with the patient's body, bodily fluids, clothes, or linen within the last 21 days. Contacts of contacts were defined as neighbours, family, or extended family members who lived within the nearest geographical boundary of all contacts, and the household members of any high-risk contacts.^3^, ^4^ High-risk contacts were defined as in the Ebola ça Suffit trial^3^ and included contacts who had either touched bodily fluids, bed linen, clothes, or dishes; had been in direct physical contact; or had slept or ate in the same household as the index case.

Another team assessed the enumerated contacts of contacts eligibility. All contacts of contacts were eligible to receive vaccination, except those individuals who were pregnant, breastfeeding, younger than 6 years of age, severely ill or immunocompromised, or had a history of anaphylaxis following vaccination. Because the vaccine was not yet licensed, written informed consent was obtained from contacts of contacts before vaccination, using thumbprint signatures if illiterate following oral explanation, and countersignature by a literate, independent witness.

### Procedures

Vaccination was provided to eligible individuals who had given consent and who were screened for eligibility. A different team assessed any immediate reactions for 30 min after vaccination. Another team visited participants at home on days 3, 14, and 21 following vaccination to document the potential occurrence of any serious adverse events. On days 3 and 14 after vaccination, we obtained information about any type of adverse event using a standardised questionnaire. We followed up all individuals with adverse events until recovery.

Current cold chain requirements need the vaccine to be kept at −80°C. The cold chain was organised from a logistical base in Conakry. Vaccine and cold chain materials were sent in several deployments by a dedicated helicopter with a trained logistician to supervise the transport. The passive vaccine storage device containing the vaccine (Arktek, Intellectual Ventures, Bellevue, WA, USA) was sent together with two additional Arktek devices without vaccine as back-up systems.

### Role of the funding source

The sponsor of the study had no role in study design, data collection, data analysis, data interpretation, or writing of the report. The corresponding author had full access to all the data in the study and had final responsibility for the decision to submit for publication.

## Results

**Figure 1:**
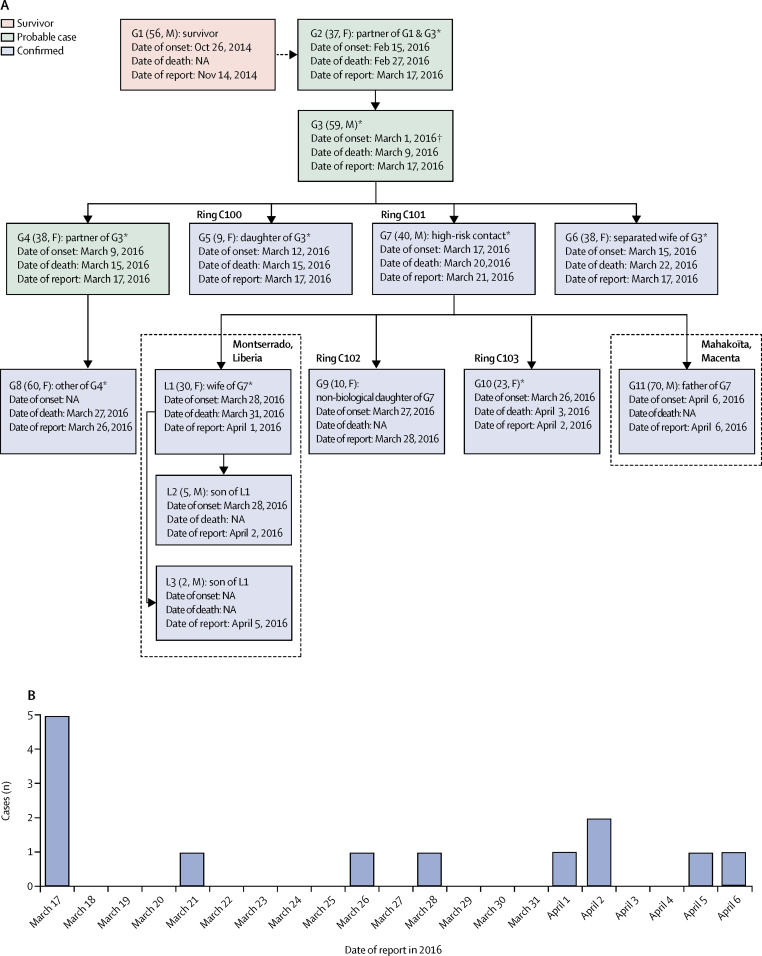
Probable chain of transmission in Guinea, from February to April, 2016 (A) Chain of transmission. (B) Date of confirmed cases refers to date of an RT-PCR positive diagnosis. Reproduced from Diallo and colleagues,^5^ by permission of Oxford Journals. G=case in Guinea. L=case in Liberia. M=male case. F=female case. NA=not available. *Died. †Possible date of onset.

Following reports of the two confirmed cases of Ebola virus disease in Nzérékoré prefecture on March 17, 2016, the national Ebola virus surveillance system identified three additional probable cases of Ebola virus disease in the area with disease onset dates between Feb 15 and March 9, 2016 (figure 1). In total, seven confirmed cases of Ebola virus disease were reported during this flare-up in Guinea, with three suspected cases identified retrospectively (figure 1). Furthermore, three other cases were reported in the Montserrado district in Liberia (figure 1).

Subsequent epidemiological investigations suggested that the assumed primary case in the flare-up, a 37-year-old woman (G2 in figure 1; disease onset was on Feb 15, 2016, and she died on Feb 27, 2016), might have been infected via sexual transmission from a male survivor of Ebola virus disease (G1) 14 months after being discharged from acute care.^5^

Of the two confirmed cases reported on March 17, 2016 (G5 and G6), who were members of the same family, the case with the earliest disease onset date was specified as the index case (G5; a 9-year-old girl with disease onset on March 12, 2016, and who died on March 15, 2016), and ring vaccination commenced on March 23, 2016 (figure 1). Another member of the family (G8; a 60-year-old woman with unknown date of onset who died on March 27, 2016) had Ebola virus disease confirmed on March 26, 2016, but was not included in the ring because she was not identified by the investigators at the time of the ring definition (figure 1).

**Figure 2:**
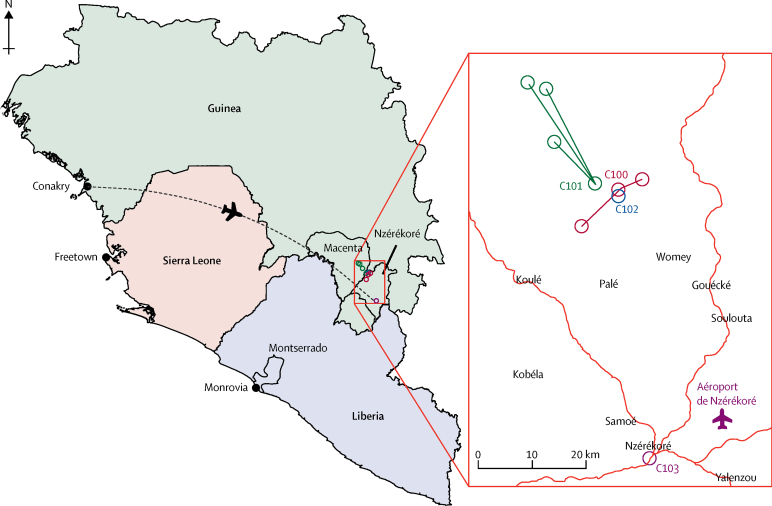
Map of west Africa and location of Nzérékoré prefecture The flight path indicates how the vaccination teams travelled from Conakry to Nzérékoré. The inset map indicates the location of the four rings defined by their index cases and their contacts and contacts of contacts, including those resident in satellite sites.

Three further confirmed cases were reported in Nzérékoré and three more rings were defined, including one with satellite sites in the neighbouring prefecture of Macenta (Guinea; figure 2). The reconstructed chain of transmission also suggests that G7's infection led to one additional case in Mahakoïta, Macenta prefecture, and three cases in Montserrado district, Liberia.

Ring vaccination was readily accepted except in one area in Macenta (Mahakoïta). Here representatives had initially accepted the compassionate use of the vaccine. However, hesitancy spread from a neighbouring village towards the Ebola virus surveillance teams, which led this community to reject the offer of vaccination, and no vaccination was implemented in this area. No further cases were detected in connection with the Mahakoïta case. Rings were not defined for G2, G3, G4, and G6 because more than 60% of its contacts and contacts of contacts were included in previous rings.

In total, 1659 contacts of contacts in Guinée Forestière were enumerated in four rings (table 1). Among the listed contacts of contacts and after screening, 1510 (91%) were eligible for vaccination (table 1). All the eligible contacts of contacts gave consent to be vaccinated and were subsequently vaccinated. Vaccinees included 303 individuals (20%) aged between 6 years and 17 years and 307 front-line workers (20%; table 1).

**Table 1:** Characteristics and individual demographics of the four vaccination rings during the Guinea outbreak response

			**C100**	**C101**	**C102**	**C103**
**Index cases used to define rings**						
Sex			Female	Male	Female	Female
Age (years)			9	40	10	23
Date of symptoms onset			March 12, 2016	March 17, 2016	March 27, 2016	March 26, 2016
Time from onset (days)						
	To admission		5	NA	0	7
	To confirmation		5	4	0	7
	To inclusion		10	8	1	8
Status at inclusion			Alive	Dead	Alive	Dead
Localisation			Rural	Rural	Rural	Urban
**Characteristics**						
Rings						
	Contacts of contacts		715	75	484	385
	Sex					
		Male	436 (61%)	60 (80%)	251 (52%)	200 (52%)
		Female	279 (39%)	15 (20%)	233/484 (48%)	185 (48%)
	Age (years)		32 (19)	44 (22)	21 (18)	33 (13)
	Age group					
		0–5 years	82 (11%)	4 (5%)	51 (11%)	0
		6–17 years	112 (16%)	6 (8%)	170 (35%)	21 (5%)
		≥18 years	521 (73%)	65 (87%)	263 (54%)	364 (95%)
	Satellite sites		2	3	0	0
	Household size		7 (4)	7 (4)	7 (4)	8 (6)
	Adults per household		2 (1)	2 (1)	2 (1)	2 (1)
Vaccinees						
	Vaccinated		632/715 (88%)	68/75 (91%)	425/484 (88%)	385/385 (100%)
		Children (6–17 years)	111/632 (18%)	5/68 (7%)	166/425 (39%)	21/385 (5%)
		Front-line workers	91/632 (14%)	3/68 (4%)	52/425 (12%)	161/385 (42%)
	Time from inclusion to vaccination (days)		5·0 (2·7)	0·5 (0·6)	3·0 (1·2)	1·4 (1·1)
	Contact with index case					
		Contact of contact	462/632 (73%)	63/68 (93%)	407/425 (96%)	339/385 (88%)
		Contact	170/632 (27%)	5/68 (7%)	18/425 (4%)	46/385 (12%)
		High-risk contact	170/632 (27%)	5/68 (7%)	18/425 (4%)	44/385 (11%)
	Compliance with follow-up visits for safety monitoring					
		Day 3	621/632 (98%)	67/68 (99%)	387/425 (91%)	382/385 (99%)
		Day 14	602/631 (95%)*	63/68 (93%)	410/425 (96%)	381/383 (99%)†
		Day 21	617/631 (98%)*	66/68 (97%)	423/425 (>99%)	381/383 (99%)†
Non-vaccinees						
	Individuals		83/715 (12%)	7/75 (9%)	59/484 (12%)	0/385 (0%)
	Reason for not being vaccinated					
		Eligible not consenting	0	2/7 (29%)	3/59 (5%)	0
		Eligible not present	0	1/7 (14%)	1/59 (2%)	0
		Not eligible (pregnant, breastfeeding, or severely ill)	0	0	5/59 (8%)	0
	Children <6 years old		82/83 (99%)	4/7 (57%)	50/59 (85%)	0
	Not listed initially		1/83 (1%)	0	0	0

Data are n, n (%), n/N (%), or mean (SD), unless otherwise stated. NA=not available.

* One serious adverse event (stroke) occurred between day 3 and day 14 after vaccination that led to hospital admission.

† Two serious adverse events (both malaria) occurred between day 3 and day 14 after vaccination that led to hospital admission.

**Figure 3:**
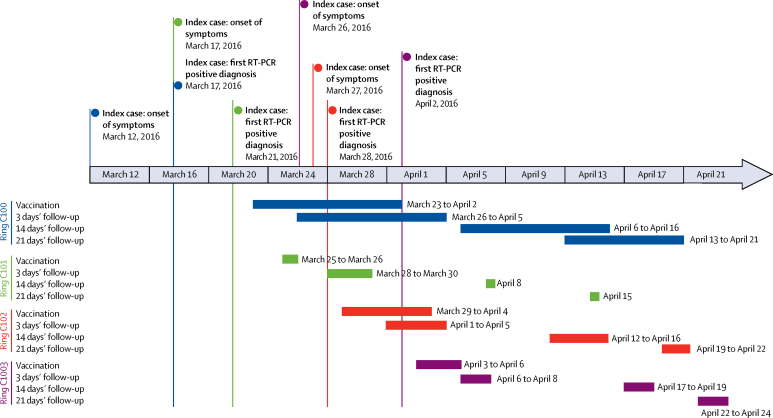
Timeline of operations in the four rings in Guinea

Although the delay from onset to confirmation of the index case was fairly consistent (between 4 days and 7 days, except for index C102, who was diagnosed on the day of onset), the median delay from confirmation of index case to vaccination of individuals in the ring decreased from 10 days to 2 days as the outbreak progressed (figure 3). This reduction in time was mainly because of the time lead associated with the first ring, which included the time to obtain protocol approval by the national authorities, to organise the transport of the teams and material to the affected site, and for the teams to engage local authorities and community.

The average size of the four rings was 414 contacts of contacts, ranging from 75 to 715, about four times larger than the size of rings defined during the ring vaccination trial. The mean age varied between rings and ranged from 21 years to 44 years. One ring (C103) was recorded as having no children younger than 6 years. All rings had a similar median household size of around eight members, including two people older than 18 years (adults). Ring C100 had two satellites and ring C101 had three satellites (figure 2).

Among eligible contacts of contacts in rings, the percentage of eligible contacts of contacts vaccinated varied from 88% to 100%. The most common reason for not being vaccinated was ineligibility because of age, with 136 (91%) of 149 ineligible contacts of contacts not vaccinated because they were younger than 6 years (table 1). Most vaccinees were contacts of contacts (between 73% and 96%) rather than contacts (table 1). By contrast, almost all contacts were high-risk contacts of the index cases (table 1).

The timings of the follow-up visits on days 3, 14, and 21 were also less variable in the later rings (figure 3). Overall, 47 6–17 year olds (17%) and 412 adults (36%) reported at least one adverse event following vaccination (table 2). Almost all adverse events occurred between 31 min and 3 days (table 2). Adults most commonly reported headache (180 [15%]), muscle pain (157 [13%]), myalgia (149 [13%]), fatigue (119 [10%]), and arthralgia (81 [7%]), which is consistent with the frequency of adverse events observed during the Ebola ça Suffit ring vaccination trial (table 2). Children aged 6–17 years most commonly reported headache (34 [12%]), muscle pain (ten [4%]), and myalgia (nine [3%]) in the overall analysis (table 2). Arthralgia symptoms were reported by one vaccinee (<1%) aged 6–17 years compared with 81 vaccinees (7%) aged 18 years or older (table 2). Three serious adverse events were reported (one case of stroke, one malaria case, and one case diagnosed with both salmonellosis and malaria), which did not show any particular sign at the day 3 follow-up visit, and did not exhibit particular clinical manifestation associated with the rVSV-ZEBOV vaccine. We concluded that no severe adverse events were causally related to the vaccine (appendix).

**Table 2:** Frequency of adverse events by time since vaccination

	**0–30 min**		**31 min to 3 days**		**4–14 days**		**Overall (0–14 days)**	
	Response rate	Frequency	Response rate	Frequency	Response rate	Frequency	Response rate	Frequency
**Adults (≥18 years, n=1207)**								
Arthralgia	100%	0	97%	80 (7%)	96%	1 (<1%)	93%	81 (7%)
Diarrhoea	100%	0	97%	3 (<1%)	96%	0	93%	3 (<1%)
Fatigue	100%	1 (<1%)	97%	118 (10%)	96%	1 (<1%)	94%	119 (10%)
Fever	100%	0	97%	1 (<1%)	96%	0	93%	1 (<1%)
Headache	100%	0	97%	180 (15%)	96%	0	94%	180 (16%)
Induration	100%	0	97%	1 (<1%)	96%	0	93%	1 (<1%)
Injection pain	100%	1 (<1%)	97%	37 (3%)	96%	0	93%	38 (3%)
Muscle pain	100%	0	97%	157 (13%)	96%	0	94%	157 (14%)
Myalgia	100%	0	97%	149 (13%)	96%	0	94%	149 (13%)
Vomiting	100%	0	97%	2 (<1%)	96%	0	93%	2 (<1%)
Other adverse events	100%	2 (<1%)	97%	29 (2%)	96%	2 (<1%)	93%	33 (3%)
Any adverse events*	100%	3 (<1%)	97%	408 (35%)	96%	4 (<1%)	94%	412 (36%)
**Children (6–17 years, n=303)**								
Arthralgia	100%	0	94%	1 (<1%)	>99%	0	93%	1 (<1%)
Diarrhoea	100%	0	94%	2 (1%)	>99%	0	93%	2 (1%)
Fatigue	100%	0	94%	3 (1%)	>99%	1 (<1%)	93%	3 (1%)
Fever	100%	0	94%	0	>99%	0	93%	0
Headache	100%	0	94%	33 (12%)	>99%	1 (<1%)	93%	34 (12%)
Induration	100%	0	94%	0	>99%	0	93%	0
Injection pain	100%	0	94%	0	>99%	0	93%	0
Muscle pain	100%	0	94%	10 (4%)	>99%	0	94%	10 (4%)
Myalgia	100%	0	94%	9 (3%)	>99%	0	94%	9 (3%)
Vomiting	100%	0	94%	0	>99%	0	93%	0
Other adverse events	100%	0	94%	3 (1%)	>99%	1 (<1%)	93%	4 (1%)
Any adverse events*	100%	0	94%	47 (17%)	>99%	2 (1%)	94%	47 (17%)

Data are % or n (%). Response rates were used to calculate the percentage of events for each line.

* Response rate calculated as the proportion of individuals who answered all adverse event fields or reported at least one adverse events.

After each ring completed vaccination, no cases were reported neither before nor 10 or more days after vaccination (figure 3). These findings are consistent with the final results of the ring vaccination trial,^6^ which showed no infection in vaccinees once 10 days had elapsed after vaccination. Moreover, none of the listed but ineligible contacts, including children under 6 years old, developed Ebola virus disease. All subsequent cases of Ebola virus disease were individuals who were either not enumerated in the rings as contacts of contacts, or were infected before the outbreak was identified.

## Discussion

During March and April, 2016, more than 1500 individuals in Guinea were vaccinated under compassionate use with the rVSV-ZEBOV vaccine in response to a flare-up of cases of Ebola virus disease. No cases of Ebola virus disease were observed among the vaccinees for 10 days or longer after vaccination, consistent with the interim analysis of the ring vaccination trial.^1^ Nor were further cases identified in unvaccinated ring members once vaccination was complete. The intervention also shows that the ring vaccination strategy can be implemented rapidly and at scale even in remote rural settings.

Three serious adverse events were reported in Guinea following vaccination but were not thought to be related to the vaccine administration. This outcome is consistent with the findings of the Ebola Ça Suffit! trial^6^ in the group of individuals aged older than 18 years, in which 80 serious adverse events were reported, but only one was related to vaccination. In previous safety studies,^7^ arthralgia has been noted as a side-effect; few cases were observed during compassionate use, and were primarily among adults. None were severe and all spontaneously resolved.

The interim results of the Guinea ring vaccination trial found no infection among vaccinees 10 days or longer after vaccination.^1^ During the 2016 Guinée Forestière compassionate use ring vaccination, no confirmed cases of Ebola virus disease were among vaccinees, nor were there further cases of Ebola virus disease reported within rings once vaccination was completed. Our data suggest that compassionate use ring vaccination together with the routine Ebola virus response strategies contributed to control this outbreak.

The operational efficiency of the ring vaccination teams improved over time. This improvement was in part facilitated by an effective partnership to ensure the transport of the cold chain and the teams. In the first ring, the median time from laboratory confirmation of the index case to vaccination of contacts and contacts of contacts was 10 days, and the follow-ups at days 3, 14, and 21 overlapped; in the fourth ring, the median time to vaccination was 2 days. The follow-up visits for all rings occurred as planned and at consistent intervals.

Individuals who were pregnant, breastfeeding, severely ill, or younger than 6 years old were not eligible for vaccination and no safety data were collected from these populations. Because the rings are defined as socioepidemiological networks, eligible contacts of contacts outside the immediate geographical area were also traced for enrolment, in addition to households of any geographically distant high-risk contacts. Therefore, field operations required constant adjustments to ensure all eligible contacts of contacts were enumerated and vaccinated if they gave their consent.

Resistance to outbreak response was a common occurrence during the Ebola virus disease epidemic in Guinea and Liberia during 2014–15.^8^, ^9^ The experience in Macenta reminds us that an adequate community engagement approach is crucial to ensure successful implementation of Ebola virus vaccination activities. The availability of the preliminary results on the efficacy of the rVSV-ZEBOV candidate vaccine—which was described in the information provided to eligible contacts of contacts—might have contributed to the high acceptance of vaccination, contact tracing, and other community Ebola virus control interventions, including among children.

In Liberia, a similar vaccination approach under expanded access was adopted following the first confirmed case of Ebola virus disease on April 1, 2016. The response involved the Centre for Disease Control and Prevention, UNICEF, WHO, the PREVAIL partnership, the Expanded Program on Immunization, and community mobilisation and health promotion teams. On the basis of the Ebola ça Suffit research protocol, modified for a public health response rather than a research protocol, geographical rings were defined around the three confirmed cases. In total, 234 individuals were vaccinated including 16 contacts, 213 contacts of contacts, and five health-care workers. No cases of Ebola virus disease were reported among those individuals vaccinated and no serious adverse events were identified through a passive follow-up.

This experience on the compassionate use of the rVSV-ZEBOV candidate vaccine under expanded access suggest that ring vaccination can be rapidly and effectively implemented as part of the Ebola virus response in case of future outbreaks. Potential operational barriers including requirements for GCP compliance, written documentation of informed consent, and a −80°C cold chain can be addressed to provide vaccination to remote, rural communities as part of Ebola virus outbreak response.

The rVSV-ZEBOV vaccine was granted access to the PRIority MEdicine (PRIME) scheme by the European Medicine Agency and Breakthrough Therapy designation by the US Food and Drug Administration. To date, the rVSV-ZEBOV vaccine is not licensed and has not been prequalified by WHO. Nevertheless, should an Ebola virus disease outbreak occur before the candidate vaccine is licensed, the WHO Strategic Advisory Group of Experts on Immunization recommended that the rVSV-ZEBOV vaccine be promptly deployed under the expanded access framework, with informed consent and in compliance with GCP. Ring vaccination is the recommended delivery strategy and should be adapted to the social and geographic conditions of the outbreak areas and include people at risk including but not limited to contacts and contacts of contacts; local and international health-care and front-line workers in the affected areas; and health-care and front-line workers in areas at risk of expansion of the outbreak.

**This online publication has been corrected. The corrected version first appeared at thelancet.com/infection on November 8, 2017**

## References

[1] Henao-Restrepo AM, Longini IM, Egger M (2015). Efficacy and effectiveness of an rVSV-vectored vaccine expressing Ebola surface glycoprotein: interim results from the Guinea ring vaccination cluster-randomised trial. Lancet.

[2] WHO Liberia and Guinea discharge final Ebola patients in latest flare-up and begin 42 days of heightened surveillance. http://www.who.int/features/2016/ebola-patients-discharge/en/.

[3] Ebola ça Suffit Ring Vaccination Trial Consortium (2015). The ring vaccination trial: a novel cluster randomised controlled trial design to evaluate vaccine efficacy and effectiveness during outbreaks, with special reference to Ebola. BMJ.

[4] WHO, CDC Implementation and management of contact tracing for Ebola virus disease. http://www.who.int/csr/resources/publications/ebola/contact-tracing/en/.

[5] Diallo B, Sissoko D, Loman NJ (2016). Resurgence of Ebola virus disease in Guinea linked to a survivor with virus persistence in seminal fluid for more than 500 days. Clin Infect Dis.

[6] Henao-Restrepo AM, Camacho A, Longini IM (2017). Efficacy and effectiveness of an rVSV-vectored vaccine in preventing Ebola virus disease: final results from the Guinea ring vaccination, open-label, cluster-randomised trial (Ebola Ça Suffit!). Lancet.

[7] Huttner A, Dayer J-A, Yerly S (2015). The effect of dose on the safety and immunogenicity of the VSV Ebola candidate vaccine: a randomised double-blind, placebo-controlled phase 1/2 trial. Lancet Infect Dis.

[8] Carrión Martín AI, Derrough T, Honomou P (2016). Social and cultural factors behind community resistance during an Ebola outbreak in a village of the Guinean Forest region, February 2015: a field experience. Int Health.

[9] The Lancet (2014). Ebola in west Africa: gaining community trust and confidence. Lancet.

