# Corrections

**DOI:** 10.1016/S1473-3099(17)30646-1

**Published:** 2017-12

*Gsell P-S, Camacho A, Kucharski AJ, et al. Ring vaccination with rVSV-ZEBOV under expanded access in response to an outbreak of Ebola virus disease in Guinea, 2016: an operational and vaccine safety report.* Lancet Infect Dis *2017; 17:1276-86*—Mosaka Fallah has been added to the author list, Severine Danmadji has been changed to Séverine Danmadji Nadlaou, and Honora Djidonou has been changed to Djidonou A Honora. The affiliation details and Contributors section have been updated to reflect these changes. These corrections have been made to the online version as of Nov 8, 2017, and the printed Article is correct.

