# Supplementary material for: Ring vaccination with rVSV-ZEBOV under expanded access in response to an outbreak of Ebola virus disease in Guinea, 2016: an operational and vaccine safety report
Source: Lancet Infect Dis. 2017 Dec;17(12):1276–84. doi: 10.1016/S1473-3099(17)30541-8 (PMC5700805; doi:10.1016/S1473-3099(17)30541-8)
Supplement: Supplementary appendix [file mmc1.pdf]

THE LANCET Infectious Diseases

Supplementary webappendix

This webappendix formed part of the original submission and has been peer reviewed. We post it as supplied by the authors.

Supplement to: Gsell P-S, Camacho A, Kucharski AJ, et al. Ring vaccination with rVSV-ZEBOV under expanded access in response to an outbreak of Ebola virus disease in Guinea, 2016: an operational and vaccine safety report. *Lancet Infect Dis* 2017; published online Oct 9. http://dx.doi.org/10.1016/S1473-3099(17)30541-8

Appendix 1: Severity of adverse events in adults and children.

	Mild		Moderate		Severe		Unknown	
**Adverse events**	**n**	**%**	**n**	**%**	**n**	**%**	**n**	**%**
**Adults**								
Arthralgia	79	97.5	2	2.5	0	0	0	0
Diarrhea	3	100	0	0	0	0	0	0
Fatigue	118	98.3	2	1.7	0	0	0	0
Fever	0	0	0	0	0	0	1	100
Headache	176	97.8	4	2.2	0	0	0	0
Induration	1	100	0	0	0	0	0	0
Injection Pain	38	100	0	0	0	0	0	0
Muscle Pain	154	98.1	3	1.9	0	0	0	0
Myalgia	146	98	3	2	0	0	0	0
Vomiting	2	100	0	0	0	0	0	0
Other AEs	33	100	0	0	0	0	0	0
Total	750	98	14	1.8	0	0	1	0.1
**Children**								
Arthralgia	1	100	0	0	0	0	0	0
Diarrhea	2	100	0	0	0	0	0	0
Fatigue	4	100	0	0	0	0	0	0
Headache	34	100	0	0	0	0	0	0
Muscle Pain	10	100	0	0	0	0	0	0
Myalgia	9	100	0	0	0	0	0	0
Other AEs	4	100	0	0	0	0	0	0
Total	64	100	0	0	0	0	0	0
